# I am better than I look: genome based safety assessment of the probiotic *Lactiplantibacillus plantarum* IS-10506

**DOI:** 10.1186/s12864-023-09495-y

**Published:** 2023-09-04

**Authors:** Alexander Umanets, Ingrid S. Surono, Koen Venema

**Affiliations:** 1 Centre for Healthy Eating & Food Innovation (HEFI), Maastricht University – campus Venlo, Villafloraweg 1, Venlo, 5928 SZ the Netherlands; 2 Chair Group Youth Food and Health, Faculty of Science and Engineering, Maastricht University – campus Venlo, Villafloraweg 1, Venlo, 5928 SZ the Netherlands; 3 Food Technology Department, Faculty of Engineering, Bina Nusantara University, Jakarta, 11480 Indonesia

**Keywords:** *Lactiplantibacillus plantarum*, Genome, Safety assessment, Comparative genomics

## Abstract

**Background:**

Safety of probiotic strains that are used in human and animal trials is a prerequisite. Genome based safety assessment of probiotics has gained popularity due its cost efficiency and speed, and even became a part of national regulation on foods containing probiotics in Indonesia. However, reliability of the safety assessment based only on a full genome sequence is not clear. Here, for the first time, we sequenced, assembled, and analysed the genome of the probiotic strain *Lactiplantibacillus plantarum* IS-10506, that was isolated from dadih, a traditional fermented buffalo milk. The strain has already been used as a probiotic for more than a decade, and in several clinical trials proven to be completely safe.

**Methods:**

The genome of the probiotic strain *L. plantarum* IS-10506 was sequenced using Nanopore sequencing technology, assembled, annotated and screened for potential harmful (PH) and beneficial genomic features. The presence of the PH features was assessed from general annotation, as well as with the use of specialised tools. In addition, PH regions in the genome were compared to all other probiotic and non-probiotic *L. plantarum* strains available in the NCBI RefSeq database.

**Results:**

For the first time, a high-quality complete genome of *L. plantarum* IS-10506 was obtained, and an extensive search for PH and a beneficial signature was performed. We discovered a number of PH features within the genome of *L. plantarum* IS-10506 based on the general annotation, including various antibiotic resistant genes (AMR); however, with a few exceptions, bioinformatics tools specifically developed for AMR detection did not confirm their presence. We further demonstrated the presence of the detected PH genes across multiple *L. plantarum* strains, including probiotics, and overall high genetic similarities between strains.

**Conclusion:**

The genome of *L. plantarum* IS-10506 is predicted to have several PH features. However, the strain has been utilized as a probiotic for over a decade in several clinical trials without any adverse effects, even in immunocompromised children with HIV infection and undernourished children. This implies the presence of PH feature signatures within the probiotic genome does not necessarily indicate their manifestation during administration. Importantly, specialized tools for the search of PH features were found more robust and should be preferred over manual searches in a general annotation.

**Supplementary Information:**

The online version contains supplementary material available at 10.1186/s12864-023-09495-y.

## Background

Probiotics are defined as “live microorganisms, which, when administered in adequate amounts, confer a health benefit on the host”. This definition has been coined by the joined working group of the FAO and WHO in 2001 and is still accepted as a consensus nowadays [1, 2]. The FAO/WHO working group also defined characteristics to which probiotics should adhere and one of these is that the strain should be safe for consumption. The probiotic *Lactiplantibacillus* (formerly *Lactobacillus*) *plantarum* IS-10506 has been isolated from a yogurt-like product, dadih, an Indonesian traditional fermented buffalo milk of West Sumatera [3, 4]. As implied by the definition, there are clinical evidences in several studies that this strain is beneficial to the host. For instance, in a human study the strain has demonstrated enhancement of humoral immune response [5]. In addition, in both adults and children it improved atopic dermatitis scores [6, 7]. In another study the strain reduced the blood LPS level in HIV-infected children undergoing antiretroviral therapy, and showed no adverse effects on the humoral mucosa and systemic immune response [8]. The strain also was shown to increase feacal IgA and immune response in children younger than two years [3, 9]. Lastly, the strain is thought to increase the production of short-chain fatty acids in women with functional constipation [10]. Moreover, the mechanism of the strain has been studied, and in vitro experiments show properties required for probiotics, such as acid and bile tolerance, adhesion to epithelium cells, and competitiveness against pathogens [11–13]. Although the strain shows some acid and bile tolerance, its survival is enhanced when micro-encapsulated [14]. In animal models the strain has been shown to have antimutagenic activity [15], and inhibition against coliforms [16]. Moreover, it stimulated the regeneration of renal tubular cells and activated intestinal stem cells in rodent models [17, 18]. The strain shows activity on the gut-brain axis, as brain-derived neurotrophic factor, neurotrophin and serotonin transporter expression was upregulated in the brain, along with intestinal serotonin levels in rats [19]. Although it has been used for more than a decade in clinical and animal trials, even in young [3, 9] and immunocompromised children [8], its genome was never sequenced. This provided an opportunity to perform an in-depth genome investigation of a probiotic that was used safely for a long time in human trials. Our goal was to determine whether we could identify genomic signatures that could be interpreted as undesirable in a safe probiotic used in humans. This is particularly interesting in light of the new regulations by the Indonesian government requiring whole genome sequencing as part of the safety assessment of probiotics. In addition, we compared different approaches for PH gene searches, as well as their comparative genomics across all available *L. plantarum* genomes (NCBI RefSeq).

## Methods

### Genome sequencing

Genome sequencing, assembly, and annotation were performed at BaseClear (Leiden, the Netherlands; Supplementary Material 1 and Supplementary Material 2). Briefly, DNA extraction was performed using a custom lysozyme/protK/bead-beating-based protocol. DNA was dissolved in Tris buffer and checked using Agilent 4200 TapeStation System and Qubit 3.0 Fluorometer. Library preparation procedure and run mode were set accordingly to used sequencing (SQK-LSK109) and barcoding kit (EXP-NBD104) protocols. Genomic DNA sequencing was performed on a GridION flowcell FLO-MIN106 (Oxford Nanopore, Eindhoven, The Netherlands). Basecalling was performed using Guppy v5.0.13 [20] with deliverables in FASTQ format. Contigs were *de novo* assembled and corrected using Flye v2.9 [21] and polished based on ONT reads using Medaka v1.4.3 (Oxford Nanopore). The assembled contigs were annotated using Prokka 1.14.6 [22]. Assembly quality and general information, such as GC content, were assessed using QUAST [23], with *L. plantarum* strain SK151 as the reference genome. GC content and skew with additional relevant information, such as the location of genes of interest and mobile elements, were visualized using the BLAST Ring Image Generator (BRIG) [24].

### Genomic features functional overview

KEGG Orthology [25] corresponding to identified codon sequences (CDSs) was retrieved using the KEGG Automatic Annotation Server (KAAS) [26] separately for chromosome and each contig. Within KAAS, we used the GHOSTX [27] search engine with the bi-directional best hit assignment, and the standard prokaryotic gene dataset (hsa, dme, ath, sce, pfa, eco, sty, hin, pae, nme, hpy, rpr, mlo, bsu, sau, lla, spn, cac, mge, mtu, ctr, bbu, syn, aae, mja, afu, pho, ape) plus *Lactobacillus plantarum* WCFS1 and *Lactobacillus plantarum* JDM1 as the reference (although the species has been renamed *Lactiplantibacillus plantarum*, the database still has the old name *Lactobacillus plantarum*). The KEGG Orthology file (BRITE format) was summarized and visualized using the R programming environment [28]. KAAS assignments were used to create a metabolic overview and search for the presence of potentially harmful (PH) or beneficial genes. For a metabolic overview, we focused only on CDSs that were assigned to the Pathway or BRITE databases. Assignments from categories that were not relevant to prokaryotes, such as human diseases (cancer), organismal systems, and mitochondrial biogenesis, were excluded.

### Presence of potentially harmful genomic features

Several approaches were used to extensively search for the presence of antibiotic resistance genes and other PH features. First, KAAS annotation was used to identify genes that were annotated to pathways or BRITE hierarchies related to antimicrobial resistance, toxin production, virulence, and human bacterial diseases. Next, a set of bioinformatics tools specifically designed to detect AMR and other PH features within the bacterial genomes was used.

For initial screening, we employed ABRicate [29] with several provided databases (CARD [30], ResFinder [31], MEGARES [32], NCBI [33], ARG-ANNOT [34], EcOH [35], and VFDB [36]) and reported hits with at least 60% coverage and 60% identity. In addition to ABRicate, we used ResFinder [31] and AMRFinderPlus [33] to diversify our approach for detecting undesirable genes. The presence of genes responsible for the production of exogenous toxins was tested using DIAMOND [37] in ultra-sensitive alignment mode against the Database of Bacterial Exotoxins for Human (DBETH) [38], and hits with at least 80% identity and 60% coverage were reported. The results from all the employed search strategies were combined into a single non-redundant table and manually curated.

We used the online server PHAge Search Tool Enhanced Release (PHASTER) [39] to identify prophages on the *L. plantarum* chromosome.

### Comparison with other L. plantarum genomes

For genome comparison, we used all available complete genomes from the NCBI RefSeq database published between 1980 and August 2022, excluding atypical genomes as defined by NCBI [40], for a total of 180 genomes (Supplementary Material 3). Only chromosomal sequences were used for genome comparison. The average nucleotide identity (ANI) was calculated using FastANI [41] and visualized as a heat map using the ComplexHeatmaps [42] R package. Genomes closely related to *L. plantarum* IS-10506 were selected based on ANI and visually compared using BRIG. In addition, we performed a pangenome analysis of the 180 reference genomes using the Roary [43] pipeline. Before processing with Roary, the genomes were re-annotated with Prokka to standardise the input data. Based on the presence or absence of homologous CDS the core (99% ≤ strains ≤ 100%), soft core (95% ≤ strains < 99%), shell (15% ≤ strains < 95%), cloud (0% ≤ strains < 15%) and unique genes per genome were identified. CDSs were identified by Roary as homologous if protein identity was at least 95%. To visualise dissimilarities between genomes, binary distances based on the presence and absence of CDSs were calculated and used for PCoA ordination (APE [44]).

For genomic features identified as PH in *L. plantarum* strain IS-10506, we used DIAMOND alignment with ultra-sensitive settings to find homologous genes in *L. plantarum* genomes from NCBI RefSeq. Features with at least 80% identity and 60% coverage were considered to be homologous.

### Identification of mobile feature, bacteriocins and CRISPR sequences

The chromosomes of *L. plantarum* IS -10506 and the reference strains were search for the presence of insertion sequences (IS) using ISEScan [45] for detection of transposable element (TE). Results were combined in the R environment using a custom script and hits with a score of zero were considered artefacts and removed prior further analysis. Overlaps between IS regions and predicted genomic features were identified based on position within the chromosome.

The search and visualisation of hypothetical bacteriocins in the genome of *L. plantarum* IS-10506 was performed using the BAGEL4 [46] web server. For the detection of CRISPRs and Cas genes, we used the online tool CRISPRCasFinder [47].

R v 4.1.2 and package tidyverse [48] were used for data handling and visualization.

## Results

**Fig. 1:**
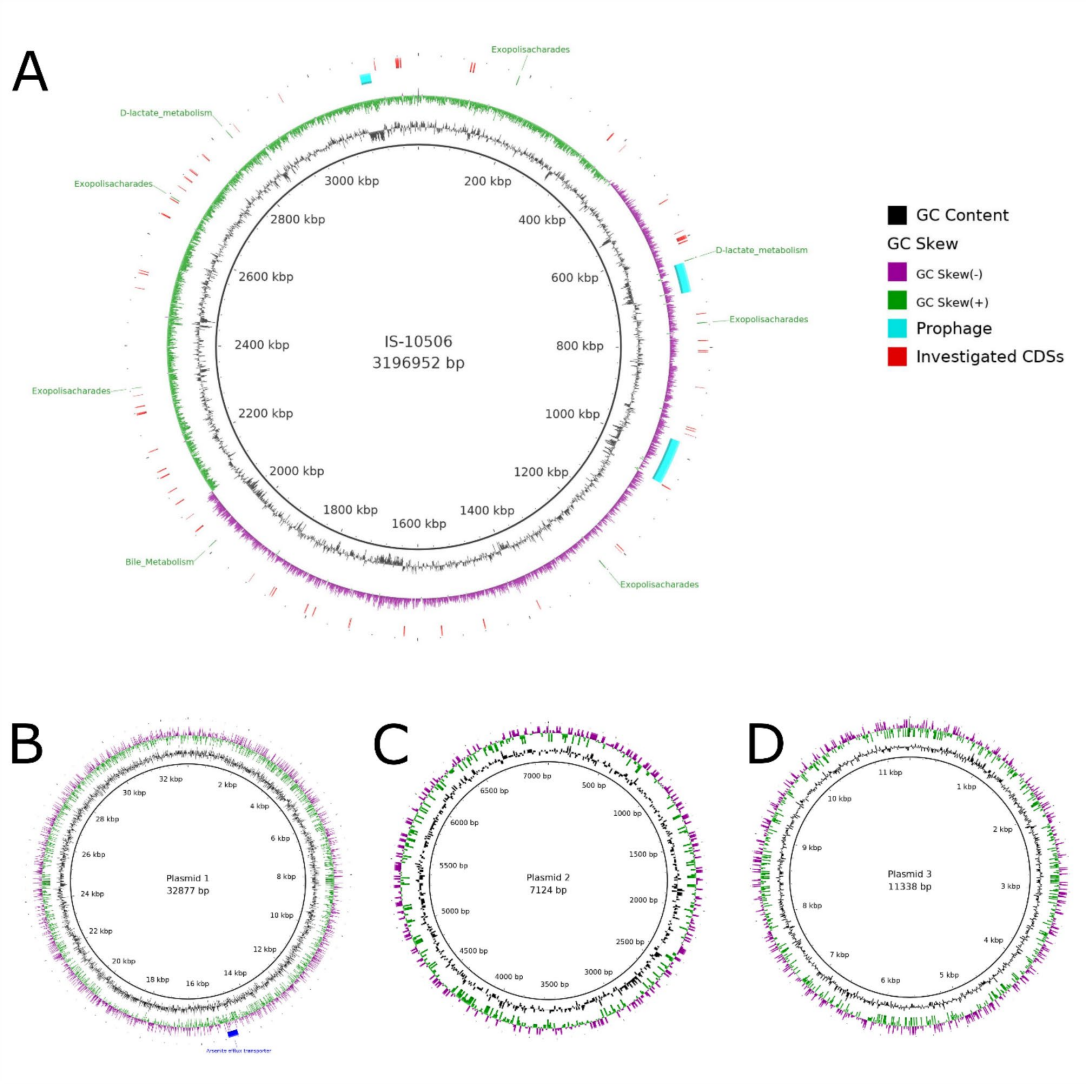
Circular representation of *L. plantarum* IS-10506 genome generated by BRIG. Figure A depicts the chromosome and figures B, C, and D the three plasmids

The assembly of the reads resulted in four contigs: one chromosome (3,196,952 bp) and three plasmids (Plasmid 1–32,877 bp, Plasmid 2–7124 bp, and Plasmid 3–11,338 bp) (Fig. 1A-D). The average coverage depth of the genome was 254 times. The GC content of the chromosome was 44.59%, with a clear definition of positive and negative strands (Fig. 1A). In total, 3059 genomic features were identified within the chromosome, of which 2975 were assigned as CDSs, 16 as rRNAs, 66 as tRNAs, 1 as tmRNA, and 1 repeat sequence. Eight, ten and 36 CDSs were identified in Plasmid 1, 2, and 3, respectively. Three prophages were identified at locations 642,325–697,269, 976,038–1,060,021, and 3,088,474–3,108,050, within the chromosome (Fig. 1A). One CRISPR sequence at location 2,199,452–2,200,081 with associated CAS-TypeIIA cluster at location 2,193,294–2,199,427 were detected with high certainty (Table S1, Supplementary Material 4).

**Fig. 2:**
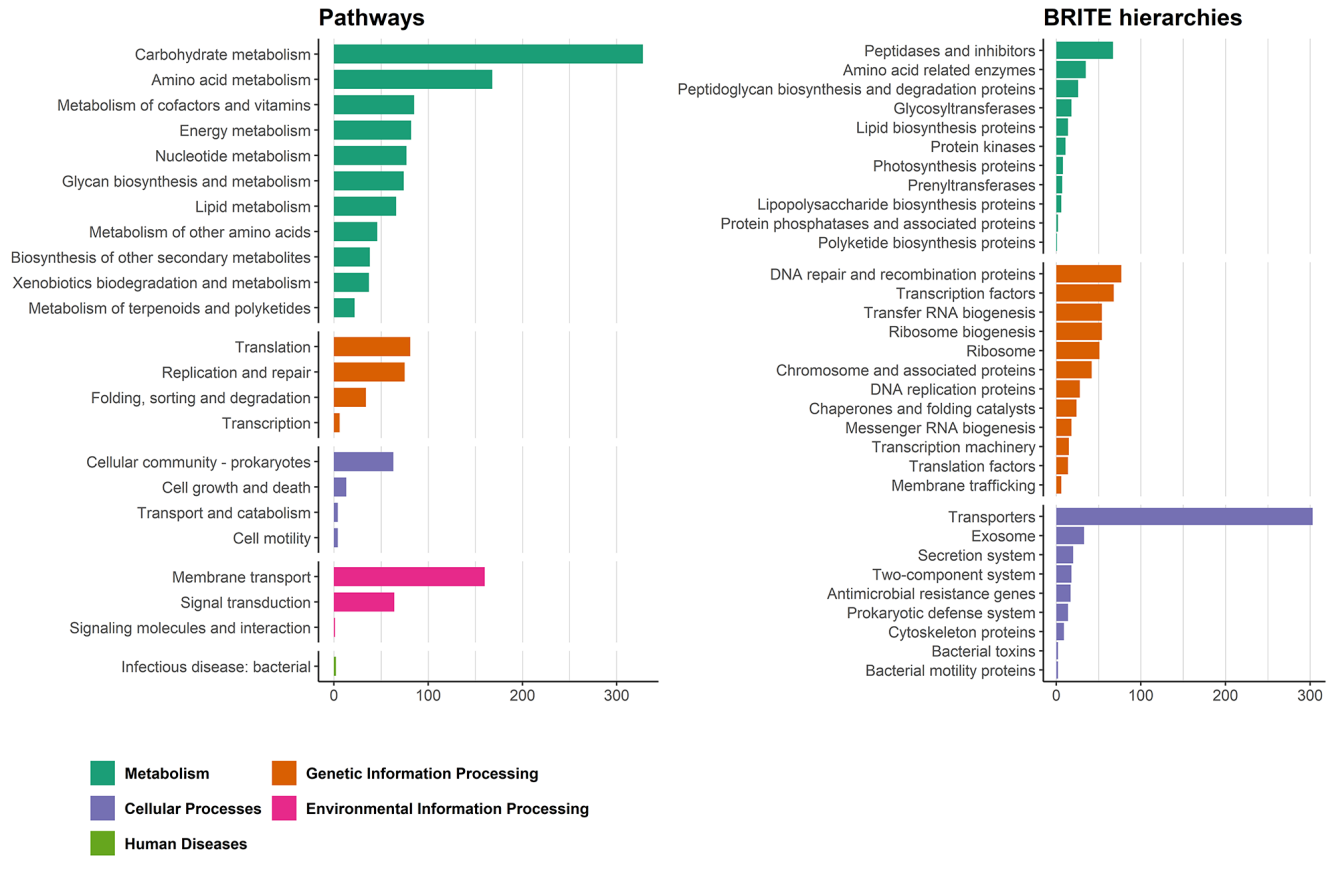
Summary of *L. plantarum* IS-10506 gene assignment by KAAS to KEGG pathways and BRITE hierarchies. The number of assigned genes is shown on the x-axis and the pathway or hierarchy group (levels B and C, respectively, in the KAAS annotation output) on the y-axis. Bar colours show the highest informative grouping of pathways or hierarchies corresponding to general functions

Out of a total of 3059 genes detected in *L. plantarum* IS-10506, only 888 could be assigned to KEGG Pathways and 925 to BRITE hierarchies using KAAS annotation servers. As expected, most of genes were assigned to metabolism and cellular or genetic information processing related pathways (Fig. 2).

Next, we focused on CDSs that belong to pathways indicated to be desirable in probiotic bacteria. Five CDSs were assigned to the pathway responsible for Exopolysaccharide (EPS) biosynthesis (KOEFPGPJ_02569, KOEFPGPJ_00158, KOEFPGPJ_00732, KOEFPGPJ_01241, KOEFPGPJ_02237), which could be beneficial for the survival of bacteria in the gastrointestinal (GI) tract. In addition, we found that one CDSs (KOEFPGPJ_01948) that was involved in pathways related to bile metabolism, which might also contribute to increased survival in the GI tract.

A number of CDSs from *L. plantarum* strain IS-10506 were assigned to KEGG pathways that could be related to potentially undesirable properties (Supplementary Material 5). However, tools designed specifically for the detection of antimicrobial resistance (AMR) and other harmful genes identified only a few of such CDSs. ABRicate identified no potential harmful (PH) genes in any database other than VFDB. Six CDSs had 66.6–72.1% identity and 65.7–93.5% coverage to homologous targets in VFDB. Three out of those six CDSs with homologues in the VFDB database were also assigned to PH pathways by KAAS: two to O-antigen nucleotide sugar biosynthesis (KOEFPGPJ_02980, KOEFPGPJ_02591), and one related to legionellosis or tuberculosis (KOEFPGPJ_02567). Interestingly, the KOEFPGPJ_01948 CDS, which has a homologous gene in VFDB, was assigned to primary and secondary bile acid biosynthesis, which could be considered a beneficial property. ResFinder identified only one CDS homologous to a disinfectant resistance gene, and annotated to chaperones and folding catalyst pathways. No AMR was found in the chromosome of IS-10506 with AMRFinderPlus, however, a CDS (KOEFPGPJ_03076) homologues to *ArsD* (arsenite efflux transporter metallochaperone) gene responsible for Arsenic resistance was identified in the plasmid sequence. We have detected the presence of two genes homologous to genes involved in bacteriocin production (*plnF* and *plnE*; Table S2, Supplementary Material 4) with BAGEL4, a tool specifically designed to detect bacteriocins. The results of BAGEL4 were consistent with the PROKKA annotation. No CDSs from IS-10506 were homologous to genes related to exotoxin production listed in the DBETH core database; however, ten CDSs had hits (80% identity and 60% coverage) against the DBETH Homologs database. According to the KAAS assignment, most of these ten CDSs are not involved in pathways related to toxin production, with the exception of KOEFPGPJ_01763 and KOEFPGPJ_01208.

**Fig. 3:**
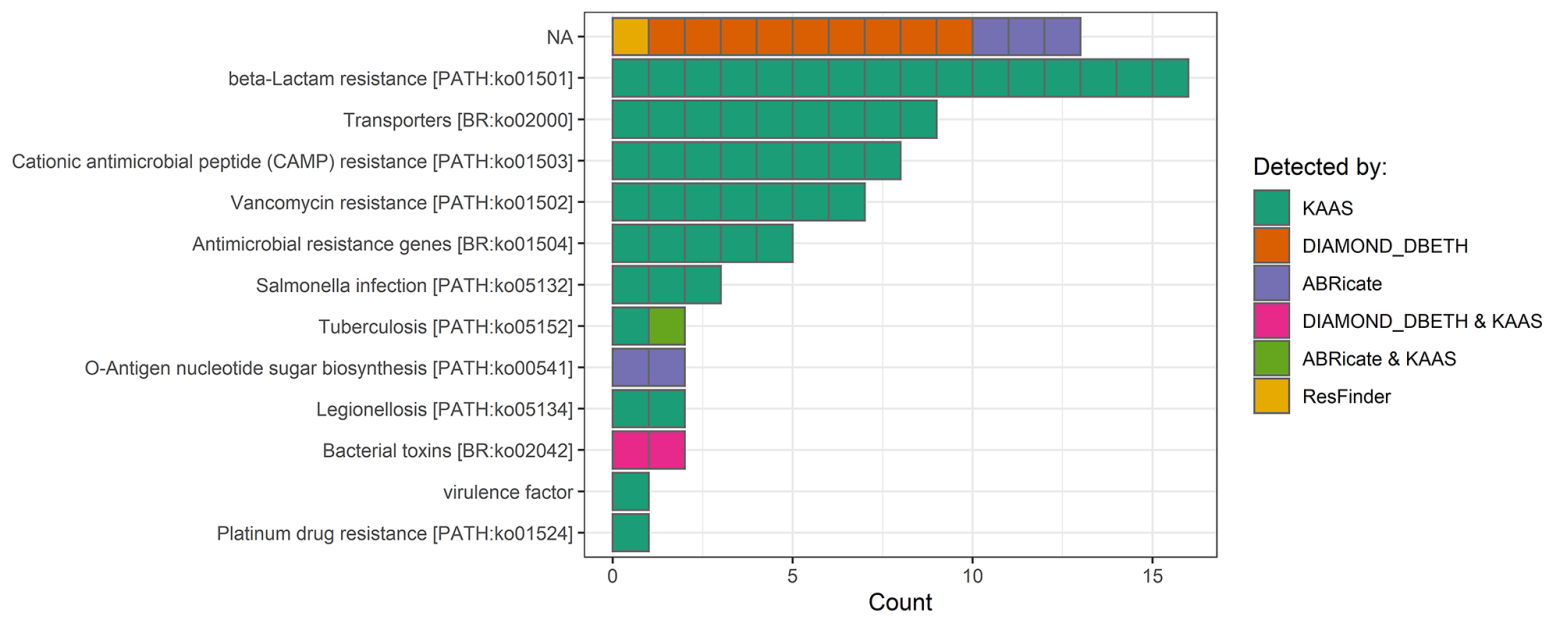
Summary of *L. plantarum* IS-10506 genes annotated as potentially harmful. The y-axis shows the metabolic annotation of a gene by KAAS; if a gene was not assigned to any potentially harmful pathway, it was assigned as not annotated (NA). Colours indicate the tool used for identification

From 58 CDSs that were assigned by KAAS to PH pathways, 16 were assigned to β-lactam resistance, 8 to cationic antimicrobial peptide (CAMP) resistance, 7 to vancomycin resistance, 5 to broad antimicrobial resistance genes, 7 related to bacterial infection (*Salmonella*, tuberculosis, and legionellosis), and 9 to AMR-related transporters (lincomycin resistance protein, multidrug resistance protein, and small multidrug resistance pump), as shown in Fig. 3. Also, two CDSs (KOEFPGPJ_00607, KOEFPGPJ_02698) were assigned as involved in D-lactate metabolism.

**Fig. 4:**
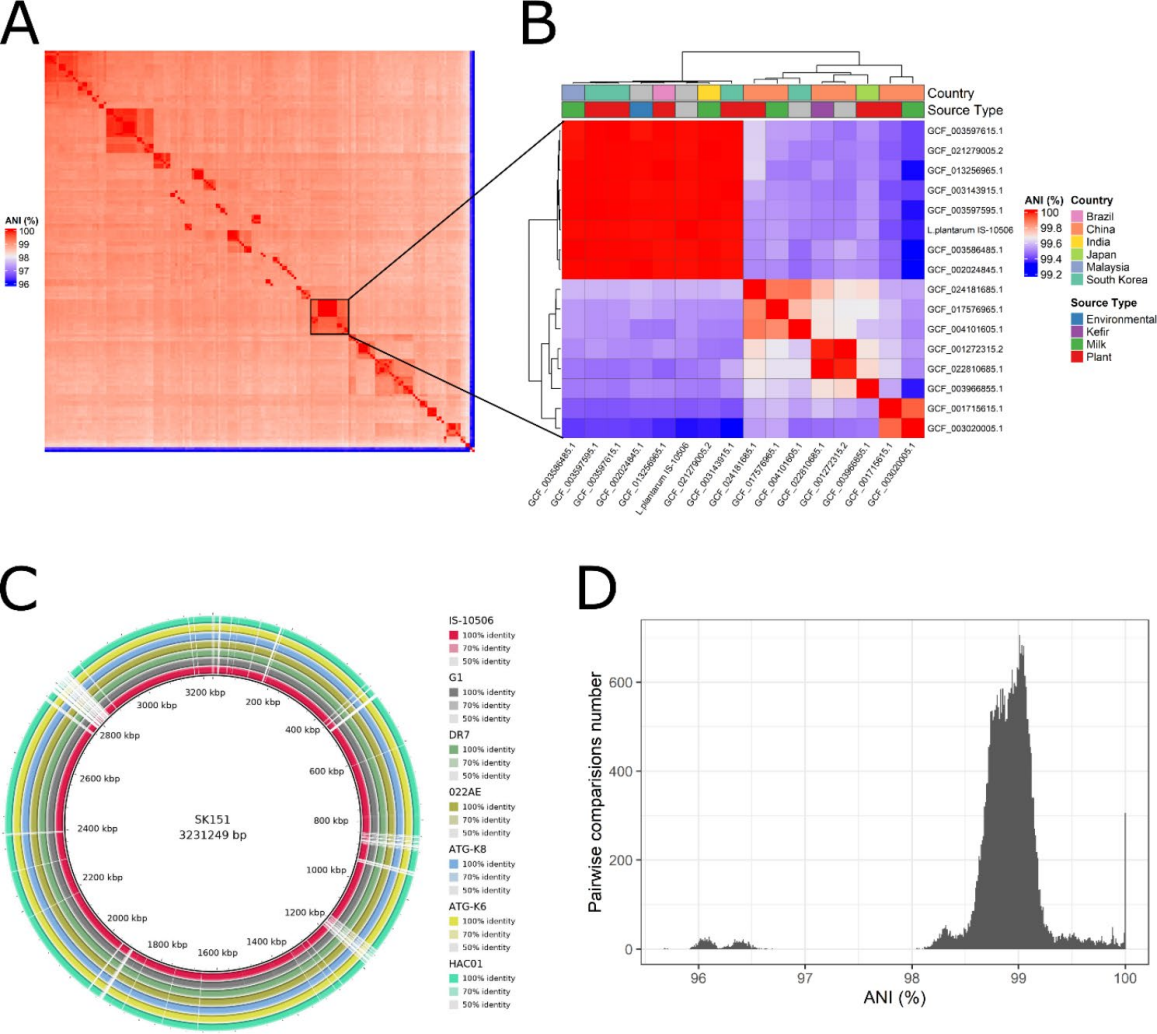
Comparison of *L. plantarum* IS-10506 with *L. plantarum* genomes from the NCBI RefSeq collection. (A) a heat-map of the average nucleotide identity (ANI) between all analyzed *L. plantarum* genomes. (B) a heat map showing only a cluster of genomes with the smallest differences in ANI from *L. plantarum* IS-10506; colored top annotation indicates strain isolation source and location. (C) a circular representation of *L. plantarum* IS-10506 genome and the six closest strains in terms of ANI genomes from NCBI RefSeq in comparison with *L. plantarum* SK151 (reference strain). (D) a histogram plotting the number of pairwise comparisons on the y-axis and the corresponding ANI on the x-axis

The overall genomes of *L. plantarum* deposited in NCBI RefSeq were very similar to each other, and most had average nucleotide identity (ANI) above 98% with a peak at 99%, and some were almost 100% identical to each other (Fig. 4A and B, and 4D). The IS-10506 genome is remarkably similar to six genomes from NCBI RefSeq, with ANI above 99.99% (Fig. 4C). The strains with these six highly similar genomes were isolated from several countries and different sources (Fig. 4A).

**Fig. 5:**
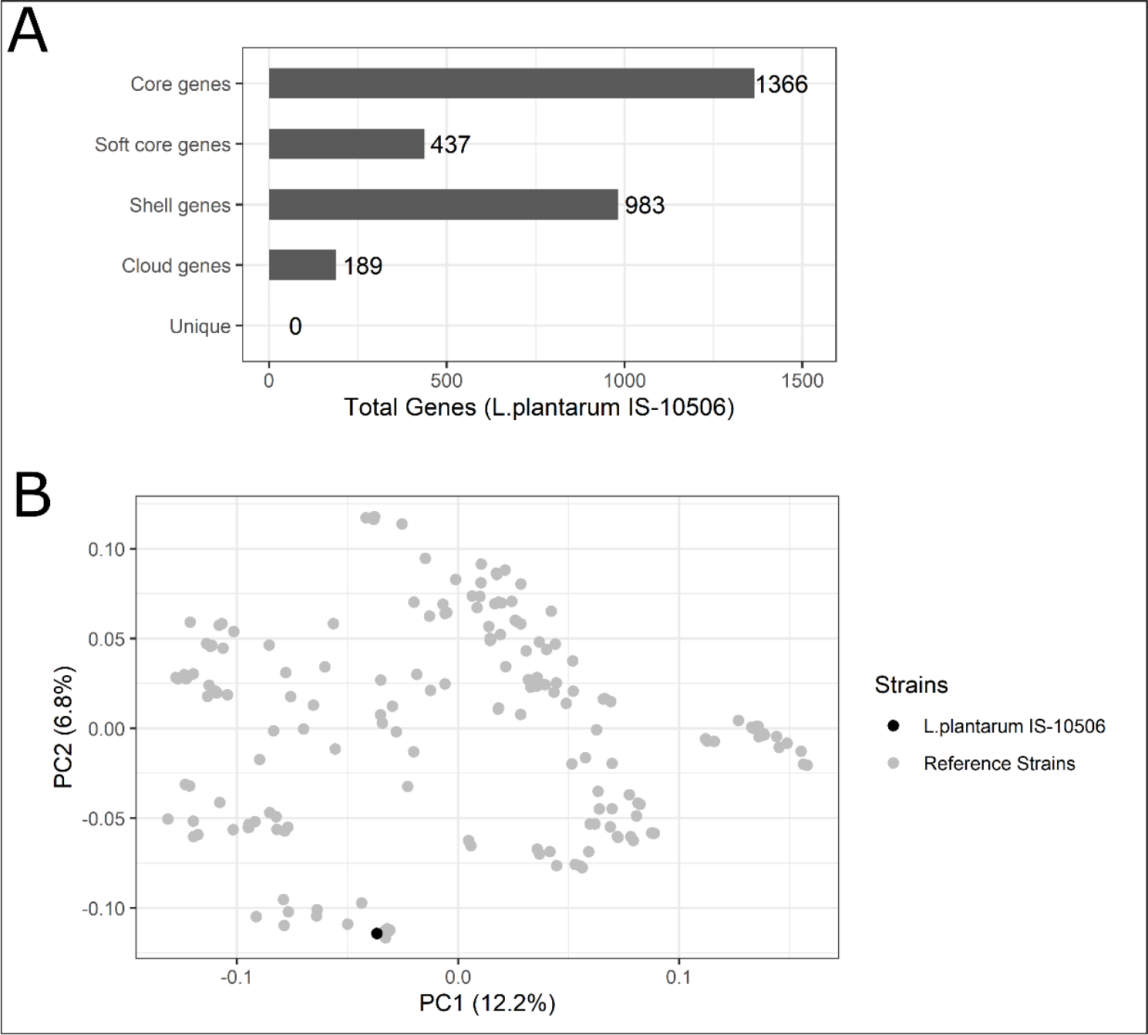
Overview of results of the *L. plantarum* pangenome analysis. Figure A shows the number of genes belonging to different gene categories within the *L. plantarum* IS -10506 chromosome. Figure B shows the first two axes of the PCoA ordination based on binary dissimilarity distances as a scatter plot

The pangenome analysis of selected *L. plantarum* strains is shown in Table S3 (Supplementary Material 4). The analysis revealed that the chromosome of *L. plantarum* IS -10506 contains no unique and only 189 cloud genes (Fig. 5A). Unsurprisingly, ordination based on gene presence-absence dissimilarity matrix shows a tight clustering of strains with high ANI identity (Fig. 5B). In the plot strain IS -10506 clusters together with the same 6 strains as in Fig. 4B and C. Of the PH genomic features, two were classified as cloud, 21 as shell, 11 as soft core and 38 as core genes (Table S3, Supplementary Material 4).

**Fig. 6:**
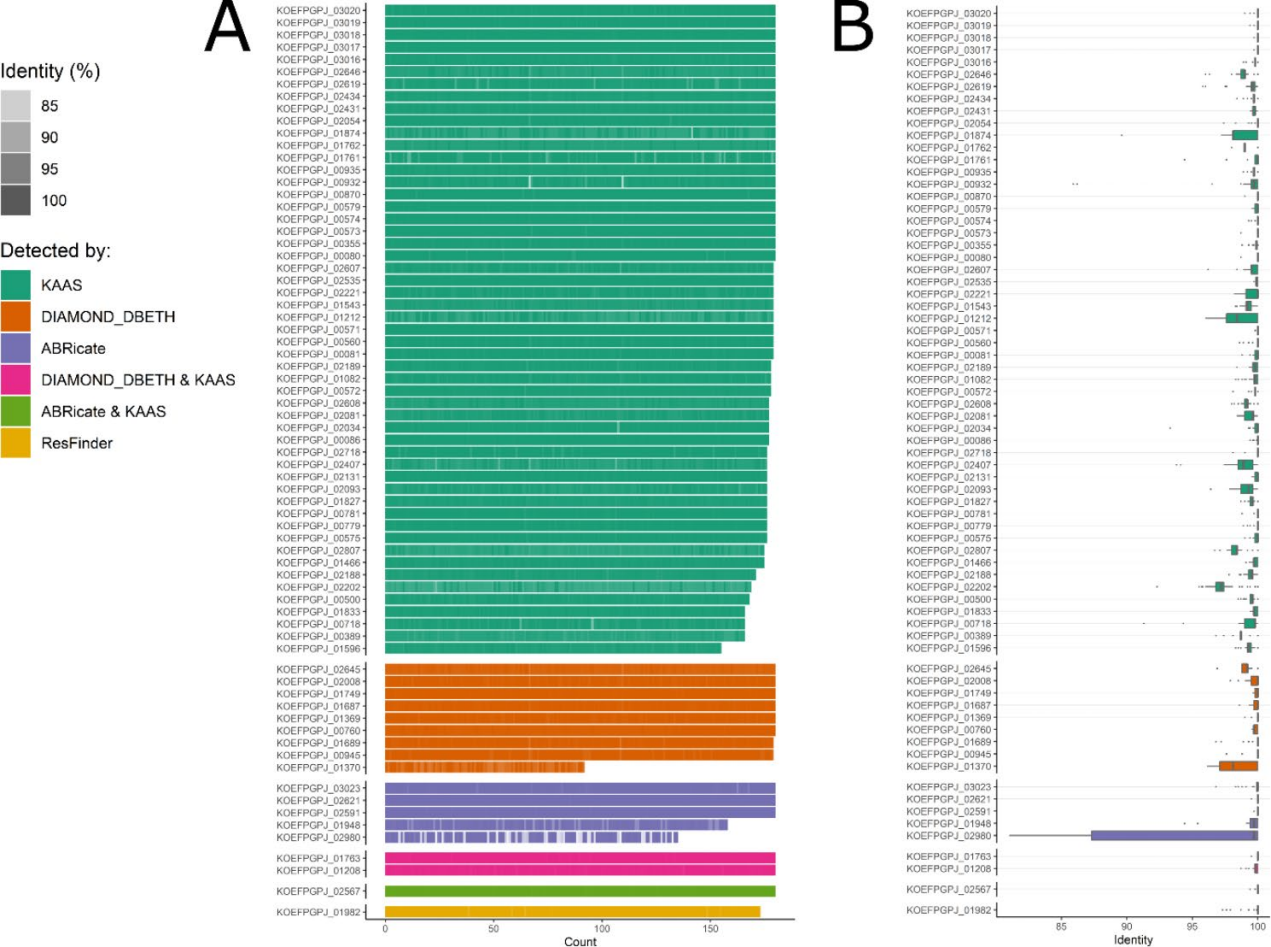
Summary of genes in *L. plantarum* genomes from the NCBI RefSeq collection homologous to potentially harmful (PH) genes identified in *L. plantarum* IS-10506. (A) number of genomes with a homologous gene on the x-axis, with the level of identity encoded as color intensity. (B) the identity of homologous genes to *L. plantarum* IS-10506 PH genes in the form of boxplots. Colours indicate PH gene detection method for both figures

We used DIAMOND search to test whether the identified PH CDSs in the *L. plantarum* IS-10506 genome have homologous genes in the *L. plantarum* genomes from NCBI RefSeq. No PH CDSs unique to the IS-10506 strain were found; moreover, out of 71 PH CDSs, 67 were found in 90% of *L. plantarum* genomes (Fig. 6A). In addition, we observed high similarity between homologous CDSs; 63 out of 71 were on average at least 99% identical (Fig. 6B). Among the CDSs with an identity score lower than 99%, the CDS KOEFPGPJ_02980 had the lowest (95.5%), followed by KOEFPGPJ_02202 (97.4%), and the remaining CDSs (KOEFPGPJ_01370, KOEFPGPJ_02807, KOEFPGPJ_00389, KOEFPGPJ_01212, KOEFPGPJ_01874, KOEFPGPJ_02407) had identity scores between 98.3% and 98.9%.

Several mobile elements have been identified in *L. plantarum* IS -10,506. Among them are three plasmids of various size. Plasmid 1 carries 36 CDSs and contains five CDSs responsible for resistance to arsenic, including pumps, transporters and a reductase (KOEFPGPJ_03073, KOEFPGPJ_03074, KOEFPGPJ_03075, KOEFPGPJ_03076 and KOEFPGPJ_03077). In addition, we observed several CDSs (KOEFPGPJ_03065, KOEFPGPJ_03089, KOEFPGPJ_03091) associated with toxin systems, namely zeta- and holin-like toxins and antitoxin from the type II toxin-antitoxin RelB/DinJ system. In plasmid 3, two of the ten CDSs were associated with toxins from the type II system PemK/MazF. Plasmid 2 was the smallest and did not appear to contain any CDSs of interest. The full description of the CDSs of the plasmids is summarised in Supplementary Material 6.

**Fig. 7:**
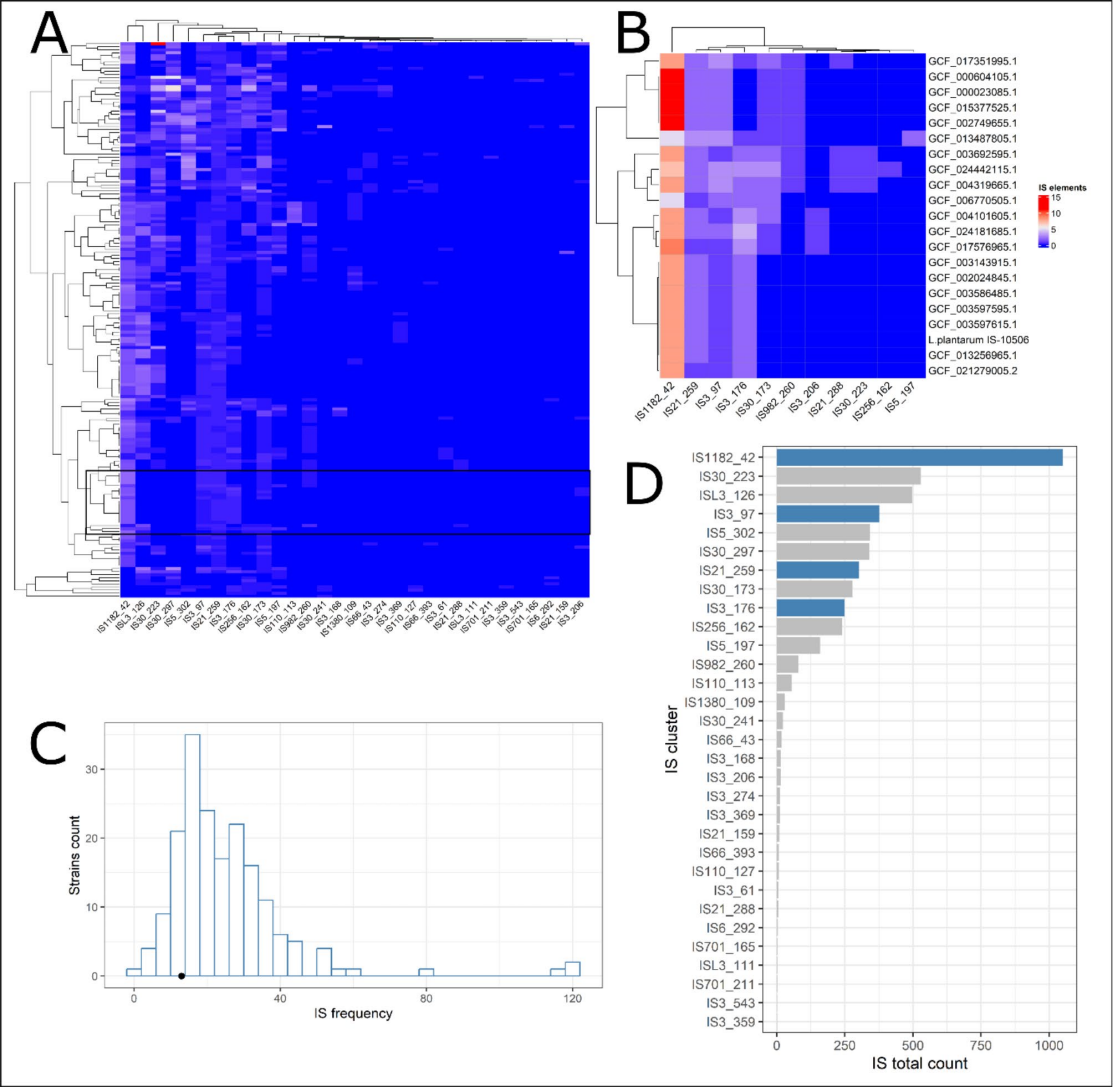
Overview of insertion sequences (IS) identified in *L. plantarum* strains (chromosomes). Figure A and B are heat maps showing the frequency of IS clusters (columns) in the investigated *L. plantarum* strains (rows); the black frame in Figure A highlights the clusters of IS shown in Figure B; rows and columns are clustered hierarchically. Figure C is a histogram showing the IS frequency (x-axis) in the *L. plantarum* strains (y-axis); the black dot on the x-axis shows the number of IS in *L. plantarum* IS -10,506. Figure D is a bar chart showing the total number of IS clusters in all *L. plantarum* strains; the bars highlighted in blue show IS clusters identified in *L. plantarum* IS -10,506

In addition to the plasmids, we identified 14 TE with four clusters of IS elements: eight copies of IS1182_42, two of IS21_259, two of IS3_176, and one of IS3_97. The *L. plantarum* IS -10,506 strain has a lower number of TE than the median of the other *L. plantarum* strains (median = 22; Fig. 7A, B and C). The IS elements identified in the chromosome of *L. plantarum* IS -10,506 were highly prevalent in all *L. plantarum* genomes examined (Fig. 7D). None of the PH genes was found in TE elements (Supplementary Material 7).

## Discussion

Despite its safe use over more than a decade in all kinds of populations, including adults [6, 10], children [5, 9] and even immunocompromised children [8], the genome of strain *L. plantarum* IS-10506 was never sequenced. This detailed investigation of the *L. plantarum* IS-10506 complete genome focused on safety and probiotic relevant features such as presence of PH genes. It was shown previously that assessment of genome safety based on the full genome sequencing is possible and a protocol was proposed previously [49]. Here we performed a synthesis analysis focusing on detection of PH genes in *L. plantarum* IS-10506 genome as well as aspects of the pan-genome analysis.

The main goal of the pan-genome analysis was to place relevant genomic features in the pan-genomic context. Overall, general results of our pan-genome analysis were very similar to comprehensive pan-genome analysis performed on 127 complete *L. plantarum* genomes in 2022 [50]. The IS-10506 genome showed a very high similarity to other genomes of *L. plantarum*, in particular six other genomes isolated from different countries (South Korea, Malaysia, India, and Brazil) and from different sources (fermented vegetables, milk, and the environment) with an ANI above 99.99%, and consequently no unique genes were identified. Such high similarities in strains isolated from different environments and continents shows that *L. plantarum* packs a versatile genetic toolkit that can be employed under different conditions. Concerning the PH genes, a high number (49 out of 71) were located in the core and soft core genes groups, providing evidences to essentiality of this genes for *L. plantarum* survival.

Mobile genetic elements are particularly important when it comes to assessment of a probiotic’s safety, due to possibility of their dissemination in the microbiome. We have identified three prophages within the *L. plantarum* – IS-10506 chromosome, however, no PH features were identified within the prophages sequences. Our findings are in line with previous reports showing extremely low prevalence of AMR genes in prophages even in complex microbial communities [51, 52]. Nevertheless, it is important to note, that AMR gene transfer between bacteria mediated by prophages is possible [53]. Plasmids are regarded as the most important mobile genetic element of bacteria and *L. plantarum* – IS-10506 carries three of them. The biggest plasmid (Plasmid 1) carries five CDS related to the arsenic resistance pathway [54]. The finding suggests the possibility of transmission of this feature to other bacteria, however, due to the high toxicity of arsenic to humans, the resistance of microbiota to it could be considered irrelevant. It is important to mention that presence of genes related to arsenic resistance was detected by general annotation, as well as, specialized tools. In addition to arsenic resistance genes, three CDSs in Plasmid 1 were homologous to genes involved associated with toxins systems. Namely, a putative holin-like toxin, a zeta toxin, and an antitoxin type II from RelB/DinJ family toxin-antitoxin system. None on the detected toxin systems is relevant to probiotic safety: antitoxin type II from RelB/DinJ family toxin-antitoxin system is related to bacterial stress response [55], holins are a diverse group of small proteins with a variety of membrane related functions [56], and zeta toxins are associated with cell death [57]. The Plasmid 3 contains two CDSs homologous to type II toxin-antitoxin system of the PemK/MazF family that is involved in bacterial cell regulatory systems [58]. The other group of mobile genetic elements is transposable elements, *L. plantarum* – IS-10506 contains a lower than average number of TEs (Fig. 7), which could suggest higher genome stability.

According to the KAAS assignment, a plethora of CDSs could potentially participate in antibiotic resistance, virulence, or toxin production. However, tools specifically designed to detect AMR and other potentially harmful genes detected only a few PH CDSs. Furthermore, while using ABRicate we had to loosen sensitivity cutoff to 60% nucleotide identity from the recommended 80% to detect any virulence factors (VFDB). Low nucleotide identity implies distant evolutionary relationships between genes, and therefore, a low chance of performing the same function. According to KEGG annotation, ABRicate-identified CDSs are homologous to genes involved in general metabolism and cellular processing, and are therefore not necessarily considered to be virulent factors. Similarly, we did not find any CDS homologous to exotoxins listed in DBETH, and only a few were listed in the DBETH homologous database. Among the CDSs with hits in the DBETH homologous database, one CDS was assigned by KAAS to the gene encoding hemolysin III, however, no hemolytic activity was observed in vitro (unpublished data). Although this finding could be a reason for concern, the same gene has been identified in other *L. plantarum* strains, including widely used probiotics [49]. It was noted that potentially virulent genes could be associated with better fitness of a strain without necessarily being harmful to the host [59]. ResFinder and AMRFinderPlus found one PH CDS each: one that was homologous to the *ClpL* gene involved in disinfectant resistance and one that was homologous to the *arsD* gene involved in arsenic resistance, respectively. The *ClpL* gene is shown to be present in other *Lactobacillus* species as well as in pathogenic bacteria and expressed in response to heat shock [60, 61]. The presence of a gene that protects bacteria against adverse conditions such as heat shock could be viewed as a desirable probiotic trait. Cell viability is one of the major concerns when it comes to probiotic administration, and the presence of genes that could help cells survive the manufacturing and administration process is highly desirable.

KEGG pathway analysis revealed a large number of genes that may be involved in resistance to various antibiotics. However, the vast majority of these genes are multifunctional and involved in the general metabolic processes of a cell. We found 16 CDSs that were homologous to β-lactam resistance genes; however, none of them were homologous to *ampA-ampG* genes, which are responsible for the induction of β-lactamase production, the primary mechanism of β-lactam resistance [62]. Ten of the 16 CDS mentioned above were homologous to the oligopeptide transport system (*OppA-OppF*), which is a part of the ATP-binding cassette (ABC) family of transporters [63]. Two other CDSs were homologous to the *bmrA* (*abcA*) gene, which is also a part of the ABC transporter family and could potentially play a role in multidrug antibiotic resistance; however, it is also an integral part of cell metabolism [64]. One CDSs was homologous to the *mrcA* gene, which is responsible for the regulation of β-lactamase production in *Stenotrophomonas maltophilia* [65]. However, it is not clear what function it could perform in *L. plantarum* strains. The presence of two CDSs homologous to *penP* and one *pbp2A* is potentially concerning because of their involvement in the production of penicillinase and penicillin-binding proteins, which are primary mechanisms of β-lactam antibiotic resistance [66]. However, we identified homologs for each of these genes in almost every *L. plantarum* genome deposited in the NCBI RefSeq, including probiotics, which indicates that these genes are omnipresent and play an important role in the survival of *L. plantarum* spp. It was observed by Bucher et al. that the *penP* gene is widely spread among *Bacillus subtili*s and helps the bacterium to survive competition within the rhizosphere environment [67]. Similarly, *penP* and *pbp2A* in *L. plantarum* could help the bacterium to be competitive and survive during fermentation.

Seven CDSs were homologous to genes involved in vancomycin resistance pathways. The genes *mraY, murF, alr, ddl*, and *murG*, according to KEGG, are included in the vancomycin resistance pathway. However, their major function is the production of proteins and enzymes necessary for the formation of the microbial cell wall, and some of them are attractive targets for future antimicrobial compounds [68–72]. Therefore, it is not surprising that CDSs corresponding to the genes listed above are present in the vast majority of *L. plantarum* genomes and share a very high degree of similarity (Fig. 6A & B). In contrast, the genes *vanY* and *vanX*, to which homologous CDSs were found in the IS-10506 genome, have been mostly investigated in relation to vancomycin resistance because of their participation in the production of alternative versions of D-Ala-D-Ala peptidoglycan, which serves as the vancomycin attachment point [73, 74]. CDSs homologous to *vanY* and *vanX* genes are also present in a large number of *L. plantarum* genomes; however, they are less conserved in comparison with the *mraY*, *murF*, *alr*, *ddl*, and *murG* genes (Fig. 6A & B). However, in the Kirby-Bauer disc diffusion method the IS-10506 strain showed a halo of 11 cm (unpublished data), and in contrast to many *L. plantarum* strains shows intermediate resistance rather than intrinsically resistant to vancomycin [75].

We found six CDSs homologous to the genes responsible for bacterial resistance to cationic antimicrobial peptides (CAMPs). CAMPs occur naturally in the environment, and a wide range of bacteria have mechanisms of self-protection against them [76]. Therefore, the relevance of CAMPs resistance genes within a probiotic genome is quite low because most probiotics come from a complex environment and have to compete with other microorganisms.

Multidrug resistance is often associated with the presence of specific transporter genes. We found that the IS-10506 genome contained several CDSs homologous to transporter genes linked to multidrug resistance. Notably, we identified several CDS copies of a single *mrs* gene. The *mdtG* gene has three homologous CDSs; *lmrB* and *emrE* have two homologous CDSs each in the IS-10506 genome. Amplification of antibiotic resistance genes can be associated with increased resistance [77]. Therefore, it is particularly important to test the antibiotic resistance of a potential probiotic strain in vitro under various conditions. However, in line with other identified PH CDSs, these multidrug resistant CDSs are present in almost all *L. plantarum* genomes, including other probiotics, and are highly conserved, indicating that these genes are important for the bacterium to survive competition in various environments.

Several genetic features are considered to be desirable for probiotics. It was found that strain IS-10506 has five CDSs that are involved in the production of EPS. EPS are a large group of diverse polymeric substances excreted by bacterial cells that are involved in various aspects of cell growth and survival [78]. Production of EPS by members of the *Lactobacillaceae* family have been explored extensively due to the fact that EPS are responsible for texture and mouthfeel of fermented products such as yogurt [79]. Therefore, the presence of genes involved in EPS production within the *L. plantarum* IS-10506 genome is not surprising, also considering its isolation source (fermented buffalo milk). In the context of probiotics and life therapeutics, EPS has been suggested to have numerous positive health effects such as reduction of cholesterol [80], modulation of intestinal immunity [81], antitumor activity [82], anti-inflammatory activity [83], and suppression of pathogens via biofilm disruption and adhesion suppression [84]. However, because of the large variation in the chemical structure of EPS, it is impossible to speculate what particular qualities and health effects are associated with EPS from IS-10506 using only genomic information. The presence of EPS-associated CDSs is a good indicator for further investigation of EPS produced by IS-10506, its structure, properties, and associated health effects, in in vitro and in vivo experiments.

Bile acids are produced in humans and animals and play a central role in lipid metabolism. They are also employed as a defence mechanism against microbial invasion, as they disrupt the bacterial membrane and lead to death of the microbial cell. Some bacteria have evolved mechanisms that suppress the activity of bile salts, by metabolizing them. On the one hand the ability to metabolize bile acids greatly increases the survivability of a bacterium in the gut environment, on the other hand microorganisms can convert bile acids into biologically active substances that can influence host signalling pathways [85]. We discovered that one CDS is homologous to the gene encoding choloylglycine hydrolase (*cbh*), and is associated with bile metabolism. Choloylglycine hydrolases convert conjugated bile salts into deconjugated bile salts, which can serve as signalling molecules and are produced by many members of the human microbiota [86]. Interestingly, the same CDSs have a distant homology to potential virulence factor in the VFDB (72% identity and 90% coverage); however, this is not surprising since the homologous potential virulence factor is the *bsh* gene that encodes bile salt hydrolase in *Listeria monocytogenes*.

We identified two CDSs that were homologous to D-lactate dehydrogenases involved in D-lactate production. D-lactate dehydrogenase is an enzyme that facilitates the reduction of pyruvate to D-lactate and has been found to be present in several species of *Lactobacillus* /*Lactiplantibacillus* [87–90]. D-lactate production by microorganisms is known to cause D-lactate acidosis, a rare neurological disease in individuals with short bowel syndrome as well as in ruminants [91]. Production of D-lactate by a probiotic strain could be viewed as an undesirable property, particularly in light of a case report of D-lactate acidosis in an infant with short bowl syndrome induced by the use of probiotics [92]. However, in healthy people, D-lactate is also produced in the GI tract by lactobacilli, bifidobacteria and other members of the endogenous microbiota, and is used as a substrate by some members of the microbiota in cross-feeding, and converted to short-chain fatty acids [93]. The amount of D-lactate produced by the gut microbiota is in large excess compared to the amounts that a probiotic could produce in the gut (Venema, unpublished results). In addition, genome analysis does not provide an understanding of the ratio of the D- to L-lactate isomers produced, which should be tested in vitro.

Ability to produce bacteriocins and presence of CRISPR inserts could give a probiotic strain competitive edge during the colonization process. Bacteriocins are small peptides that inhibit or kill with usually a small spectrum of actvitiy, mostly killing closely related bacteria of the same species or genus. We identified two genes involved in plantaricin F and E (*plnF* and *plnE*) production. Plantaricins are well studied bacteriocins produced by a variety of *L. plantarum* strains and help them compete in their environment, e.g., during the fermentation process [94]. We have identified only one type II CRISPER-Cas system that is fairly common among bacteria in general and *L. plantarum* strains in particular [95].

## Conclusion

For the first time, the genome of *L. plantarum* probiotic strain IS-10506 was fully assembled, analysed, and compared with other *L. plantarum* strains. A thorough investigation of the IS-10506 genome revealed several potential points of concern, such as the presence of AMR genes and genes involved in D-lactate production. However, in all cases, homologous genes were found across the majority of *L. plantarum* genomes, including other commercially available probiotics, indicating their importance in the core metabolism and survival of the bacterium in the environment. Moreover, these genes were inferred from the KEGG annotation rather than identified by specialized tools, showing the superior performance of specialized tools in comparison with the general annotation approach. The actual resistance of the IS-10506 strain should be tested using in vitro assays, but its safe use in numerous clinical trials without any reported adverse effects shows that the results of genome based safety assessment and real world application results are not completely mirroring each other.

### Electronic supplementary material

Below is the link to the electronic supplementary material.

Supplementary Material 1: Complete nucleotide sequence of the *L. plantarum* IS-10506 genome.

Supplementary Material 2: General annotation of the *L. plantarum* IS-10506 genome.

Supplementary Material 3: List of 180 *L. plantarum* genomes used to determine the pan-genome.

Supplementary Material 4: Supplementary tables: Table S1. Genes included in the CAS cluster identified within *L. plantarum* IS -10506 genome; Table S2. Bacteriocin producing genes identified within *L. plantarum* IS -10506 genome; TableS3. Pan-genome analysis summary of table. The table shows number of genes per gene group.

Supplementary Material 5: Summary of potentially harmful genes.

Supplementary Material 6: CDSs detected in plasmids.

Supplementary Material 7: Identified insertions sequences flanking transferable elements in *L. plantarum* IS-10506 genome.

## Data Availability

The assembled genome and annotation are available as the supplementary materials files IS10507_genome _S2.txt and IS10507_genome_annotation_S3.txt in fasta and geneBank formats respectively. Basecalled Nanopore reads are available from the NCBI bio-projects with PRJNA917529 accession ID (https://www.ncbi.nlm.nih.gov/sra/PRJNA917529).
