# Supplementary material for: I am better than I look: genome based safety assessment of the probiotic Lactiplantibacillus plantarum IS-10506
Source: BMC Genomics. 2023 Sep 4;24:518. doi: 10.1186/s12864-023-09495-y (PMC10478331; doi:10.1186/s12864-023-09495-y)
Supplement: Supplementary file 4 — Supplementary Material 4: Supplementary tables: Table S1. Genes included in the CAS cluster identified within L. plantarum IS -10506 genome; Table S2. Bacteriocin producing genes identified within L. plantarum IS -10506 genome; TableS3. Pan-genome analysis summary of table. The table shows number of genes per gene group. [file 12864_2023_9495_MOESM4_ESM.docx]

**Supplementary 4: I am better than I look: Genome based safety assessment of the probiotic Lactiplantibacillus plantarum IS-10506**

*Alexander Umanets, Ingrid Surono and Koen Venema*

**Table S1.** Genes included in the CAS cluster identified within *L. plantarum* IS -10506 genome.

| **Gene name** | **Genome position start** | **End** | **Orientation** |
| --- | --- | --- | --- |
| cas9_TypeII | 2,193,294 | 2,197,370 | + |
| cas1_TypeII | 2,197,565 | 2,198,470 | + |
| cas2_TypeI-II-III | 2,198,448 | 2,198,753 | + |
| csn2_TypeIIA | 2,198,750 | 2,199,427 | + |

**Table S2.** Bacteriocin producing genes identified within *L. plantarum* IS -10506 genome.

| ***pln* genes** | **Function** | **Amino acid sequence** |
| --- | --- | --- |
| plnF | Bacteriocin | MKKFLVLRDRELNAISGGVFHAYSARGVRNNYKSAVGPADWVISAVRGFIHG |
| plnE | Bacteriocin | MLQFEKLQYSRLPQKKLAKISGGFNRGGYNFGKSVRHVVDAIGSVAGIRGILKSIR |

**Table S3.** Pangenome analysis summary of table. The table shows number of genes per gene group.

| **Genes group** | **Cut-off criteria** | **Number of genes** |
| --- | --- | --- |
| Core genes | 99 % ≤ strains ≤ 100 % | 1366 |
| Soft core genes | 95 % ≤ strains < 99 % | 463 |
| Shell genes | 15 % ≤ strains < 95 % | 1774 |
| Cloud genes | 0 % ≤ strains < 15 % | 9343 |
| Total genes |  | 12946 |
